# An outbreak of SARS-CoV-2 with high mortality in mink (*Neovison vison)* on multiple Utah farms

**DOI:** 10.1371/journal.ppat.1009952

**Published:** 2021-11-12

**Authors:** Chrissy D. Eckstrand, Thomas J. Baldwin, Kerry A. Rood, Michael J. Clayton, Jason K. Lott, Rebecca M. Wolking, Daniel S. Bradway, Timothy Baszler

**Affiliations:** 1 Washington Animal Disease Diagnostic Laboratory, Washington State University, Pullman, Washington, United States of America; 2 Utah Veterinary Diagnostic Laboratory, Utah State University, Logan, Utah, United States of America; 3 Utah State University, Animal, Dairy, and Veterinary Sciences, Logan, Utah, United States of America; 4 Fur Breeders Agricultural Cooperative, Logan, Utah, United States of America; Erasmus Medical Center, NETHERLANDS

## Abstract

The breadth of animal hosts that are susceptible to severe acute respiratory syndrome coronavirus 2 (SARS-CoV-2) and may serve as reservoirs for continued viral transmission are not known entirely. In August 2020, an outbreak of SARS-CoV-2 occurred on five mink farms in Utah and was associated with high mink mortality (35–55% of adult mink) and rapid viral transmission between animals. The premise and clinical disease information, pathology, molecular characterization, and tissue distribution of virus within infected mink during the early phase of the outbreak are provided. Infection spread rapidly between independently housed animals and farms, and caused severe respiratory disease and death. Disease indicators were most notably sudden death, anorexia, and increased respiratory effort. Gross pathology examination revealed severe pulmonary congestion and edema. Microscopically there was pulmonary edema with moderate vasculitis, perivasculitis, and fibrinous interstitial pneumonia. Reverse transcriptase polymerase chain reaction (RT-PCR) of tissues collected at necropsy demonstrated the presence of SARS-CoV-2 viral RNA in multiple organs including nasal turbinates, lung, tracheobronchial lymph node, epithelial surfaces, and others. Localization of viral RNA by *in situ* hybridization revealed a more localized infection, particularly of the upper respiratory tract. Whole genome sequencing from multiple mink was consistent with published SARS-CoV-2 genomes with few polymorphisms. The Utah mink SARS-CoV-2 strains fell into Clade GH, which is unique among mink and other animal strains sequenced to date. While sharing the N501T mutation which is common in mink, the Utah strains did not share other spike RBD mutations Y453F and F486L found in nearly all mink from the United States. Mink in the outbreak reported herein had high levels of SARS-CoV-2 in the upper respiratory tract associated with symptomatic respiratory disease and death.

## Author summary

The recent emergence and spread of the novel coronavirus has resulted in worldwide disease and economic hardship. The virus, known as SARS-CoV-2 is believed to have originated in bats and has spread worldwide through human-to-human virus transmission. It remains unclear which animal species, other than humans, may also be susceptible to viral infection and could naturally transmit the virus to susceptible hosts. In this study, we describe the early phases of an outbreak of disease and death due to SARS-CoV-2 infection in farmed mink in Utah, United States. The investigation reveals that mink can spread the virus rapidly between animals and that the disease in mink is associated with viral infection and damage to tissues of the upper and lower respiratory system. The determination that mink are susceptible to SARS-CoV-2 indicates the need for strict biosecurity measures on mink farms to remediate mink-to-mink and human-to-mink transmission for the protection of mink, as well as prevent potential transmission from mink to humans. It may be prudent for humans working with SARS-CoV-2 mink to wear appropriate PPE until more is understood.

## Introduction

Since December 2019, worldwide spread of a novel coronavirus designated as severe acute respiratory syndrome coronavirus 2 (SARS-CoV-2) has resulted in significant human disease, death, and economic loss [1]. Phylogenetic evidence suggests that SARS-CoV-2 may have jumped from the Intermediate Horseshoe Bat (*Rhinolopphus affinis*) to human beings, possibly via an undetermined intermediate host [2–4]. If proven, this is an example of a generalist coronavirus broadening its host range, as occurred with the 2002 emergence of Severe Acute Respiratory Syndrome and the 2012 emergence of Middle Eastern Respiratory Syndrome (MERS) from wild bats in Saudi Arabia [5–14]. As countries continue to modify infection control and public health strategies for containment of SARS-CoV-2, sources of viral transmission from domestic and wildlife animal reservoirs are of great interest.

Experimental SARS-CoV-2 infection studies demonstrate susceptibility of rhesus macaques, cats, dogs, ferrets, mice, tree-shrews, Egyptian fruit bats, and Syrian guinea pigs to the virus with variable permissiveness and expression of clinical disease; while pigs, poultry and cattle do not appear to be susceptible [15–25]. How these experimentally susceptible animal hosts contribute to the transmission dynamics of SARS-CoV-2 in nature is yet to be definitively determined. There have been sporadic reports of natural SARS-CoV-2 infection in domestic dogs and cats, as well as large cats and great apes in zoological facilities [26–30], however mink appear to be the animal species that is most significantly impacted by SARS-CoV-2 infections.

SARS-CoV-2 infections have been reported in farmed mink worldwide, including the United States [31–36] with the index report coming from the Netherlands [32]. Full-length viral genome sequencing from mink SARS-CoV-2 outbreaks in the Netherlands and Denmark suggested novel virus variant transmission between mink and humans, and an increased potential for viral spread in this environment [33,36]. These outbreaks have led to major economical and health concerns, both for mink industry as mass culling and animal loss due to disease results in major losses, as well concerns for human health as viral transmission persists [37]. Surveillance and characterization of SARS-CoV-2 outbreaks at the human-mink interface are crucial for understanding viral transmission dynamics, viral genomic evolution, and disease pathogenesis.

In August 2020 multiple mink farms in Utah experienced a sudden increase in animal mortality attributed to natural SARS-CoV-2 infection due to significant inter-animal transmission. Subsequent to this outbreak there have been reports of SARS-CoV-2 infection on mink farms in Oregon, Wisconsin and Michigan [38]. The primary aim of this report is to describe the early stages of a SARS-CoV-2 outbreak that occurred in Utah mink farms focusing on clinical parameters (clinical disease, morbidity and mortality), pathogenesis of disease (gross and microscopic lesions, viral load, virus distribution), and viral genetic analysis. This study aims to provide information for a to better understanding of SARS-CoV-2 disease pathogenesis in naturally infected mink at the individual animal, cellular and molecular levels.

## Results

### Premise and animal information

The outbreak of disease associated with SARS-CoV-2 infection began in August 2020. Five mink farms were included in this investigation in which two farms had a common producer and the others were operated independently. Three premises had common labor between farms and were in close physical proximity (approximately 400 meters). Farms each had perimeter fences, locked gates, and access only to authorized personnel. Mink were housed in roof-covered sheds with ventilation to the outdoors through open side walls. Adult animals were held either individually or coupled in wire mesh cages with an approximately 1-inch space between cages to prevent inter-animal aggression, but nose-to-nose contact between neighboring animals was possible. Diets were comprised of offal and a carbohydrate source and were mixed in two distinct kitchens distributed daily to the five farms. Watering systems were variable between premises and animals had either individual nipple waterers, individual water dishes or a trough system. Mink were vaccinated annually against *Clostridium botulinum*, mink enteritis virus, canine distemper virus, and *Pseudomonas aeruginosa*. Aleutian mink disease virus was intermittently identified as a cause of disease on the farms and considered a possible comorbidity. Wildlife, including skunks and raccoons, were intermittently observed on the premises and eliminated on an as-needed basis. Feral cats were commonly present on premises to assist with rodent control.

**Fig 1:**
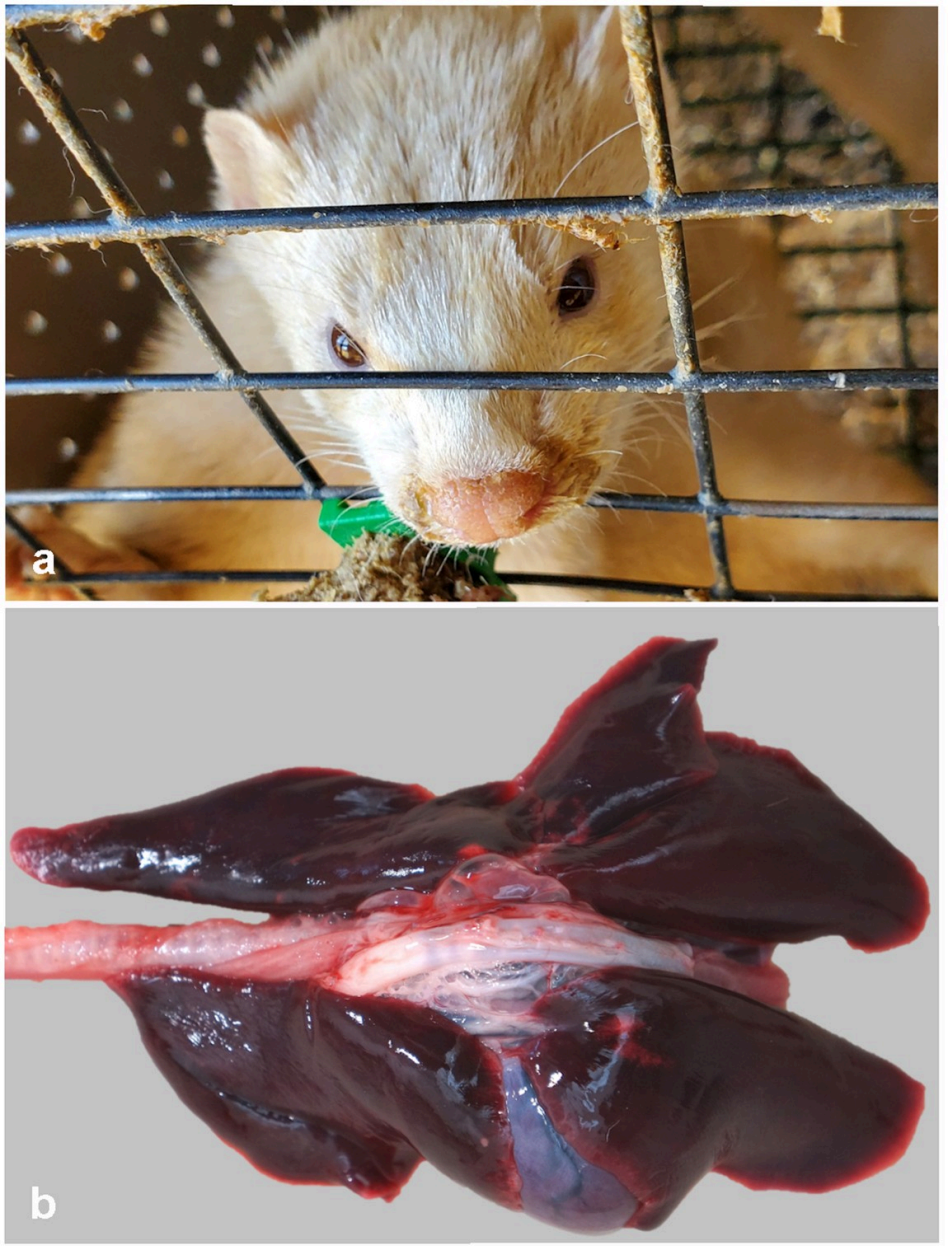
Clinical and gross necropsy findings in SARS-CoV-2 infected mink. A. A mucopurulent nasal discharge, indicative of rhinitis, stains the fur surrounding the nares in a SARS-CoV-2 infected mink. B. Gross image of severe pulmonary congestion and edema of an infected mink.

Clinical disease and death was observed in adult breeding animals ranging in age from 1–5 years, while young-of-the-year kits were overwhelmingly unaffected by the virus. The first disease indicator noted by producers was an abrupt increase in the overall mortality rate. The mortality rate ranged from 35–55% in the adult-aged mink, which normally ranges between 2 and 6%. On one premise the mortality rate in female mink was 1.8 times greater than males. An increase in respiratory effort was notable in diseased mink characterized by gasping or increased abdominal effort. Upper respiratory signs included nasal and ocular discharge (Fig 1A), and coughing was present, but was variable between farms. There was no report of gastroenteritis. Survival with resolution of respiratory disease was observed in some animals without observable lasting effects, however the frequency of this occurrence is unknown.

### Pathology

A total of 20 dead mink with minimal grossly visible postmortem decomposition, both female and male, from five farms were available for postmortem examination (necropsy). Given the clinical suspicion of SARS-CoV-2 infection and the potential risk to human prosectors, necropsies were performed with personal protective equipment in accordance with biosafety level 3 practices (conducted in Class II biosafety cabinet with appropriate primary barriers and personal protective equipment including clothing, gloves, eye, face and respiratory protection). Mink were generally in good body condition based on adipose tissue stores and muscling. In all mink, lung lobes were uniformly (most common) or variably dark red, heavy, and failed to collapse (Fig 1B). Abundant clear fluid escaped when lung lobes were incised, and tracheas contained variable amounts of white froth (pulmonary edema).

**Fig 2:**
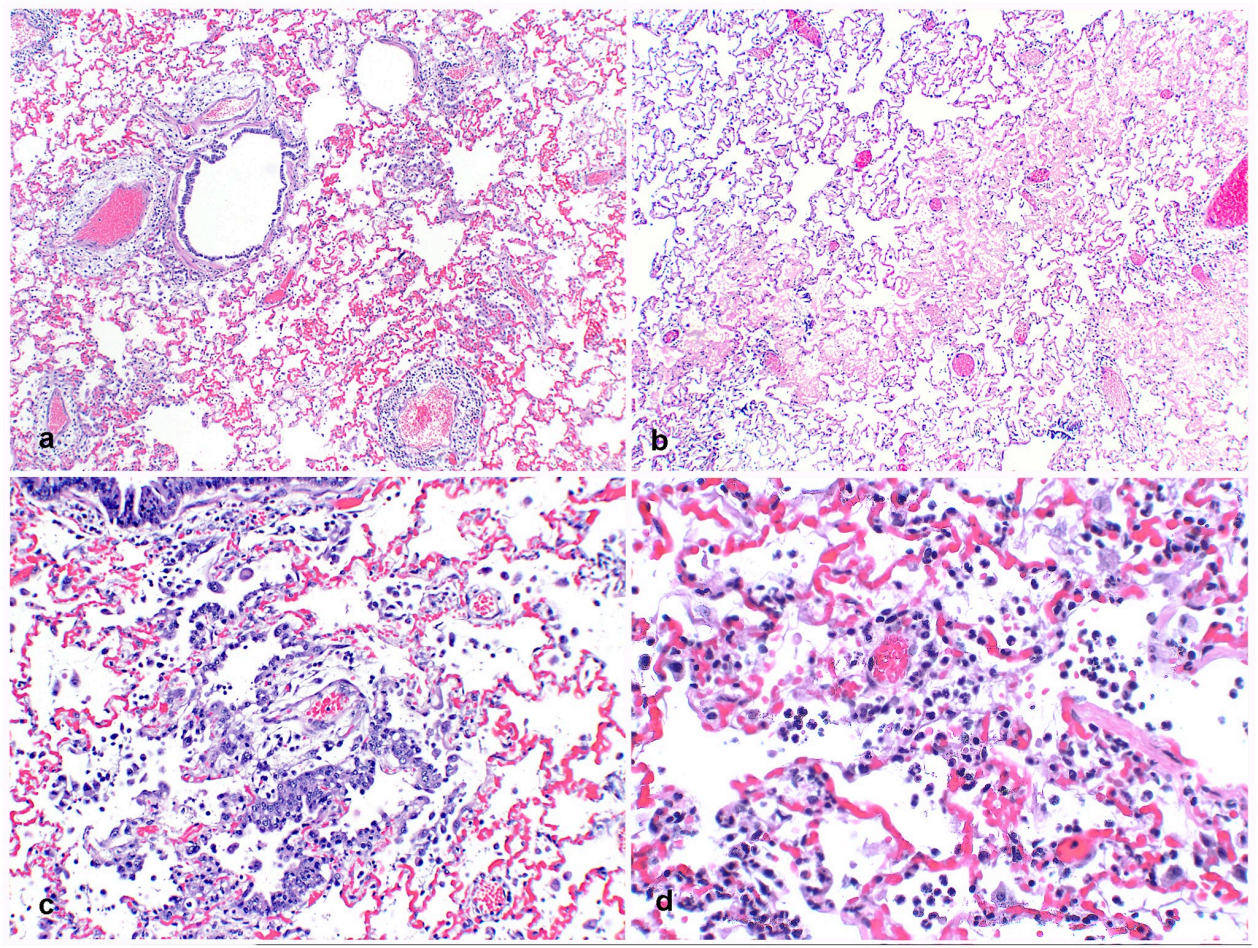
Pulmonary histopathology of SARS-CoV-2 infected mink. A. Lung from an adult mink with large cuffs of mononuclear inflammatory cells and edema multifocally surrounding pulmonary vessels. 20x H&E. B. Alveolar spaces are multifocally filled with eosinophilic edema fluid. 40x H&E. C. Bronchioles are lined with proliferative, slightly disorganized hyperplastic epithelium and type II pneumocyte hyperplasia is present in alveoli associated with increased intra-alveolar inflammation. 100x H&E. D. Neutrophils, fewer macrophages, and strands of fibrin are multifocally present in alveoli. 200x H&E.

Microscopic examination revealed multifocal interstitial and perivascular pneumonia (Fig 2A) and variable amounts of alveolar edema (Fig 2B) in all mink. Occasional fibrin strands overlay necrotic alveolar pneumocytes. Proliferative type II pneumocytes infrequently lined other alveolar septa (Fig 2C). Low to moderate numbers of neutrophils and macrophages plus moderate amounts of fibrin were in multiple alveolar spaces (Fig 2D). In nearly all pulmonary arterioles, edema fluid and moderate numbers of lymphocytes and plasma cells widely separated collagen fibers of the tunica adventitia. Sporadic vessels had mural fibrinoid degeneration. Additional findings included mild, diffuse, catarrhal to necrotizing enterocolitis (5/20 mink), moderate, multifocal, splenic lymphoid necrosis (5/20 mink), severe acute centrilobular hepatic congestion (4/20 mink), focal perivascular lymphocytic meningitis (1/20 mink), severe necrotizing and suppurative bridging centrilobular hepatitis (1/20 mink) and myocardial interstitial fibrosis and fatty infiltration (1/20 mink). Severe suppurative rhinitis with multifocal attenuation and loss of epithelial cells was noted by microscopic examination of nasal turbinates from two mink (2/20 mink).

### SARS-CoV-2 RT-PCR and tissue distribution

**Fig 3:**
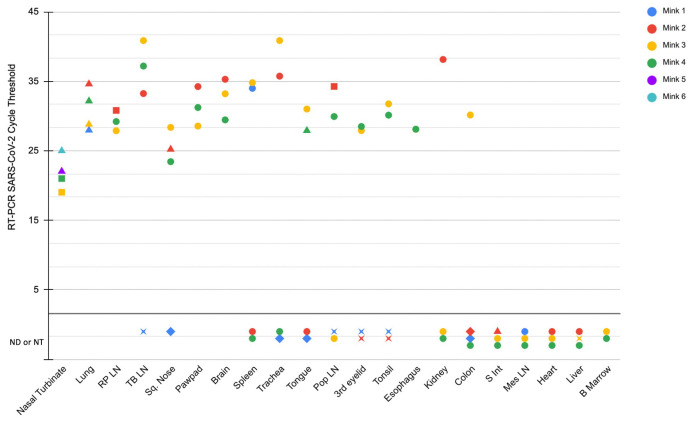
Detection of SARS-CoV-2 RNA in tissues by RT-PCR and ISH. Tissues where viral RNA was not detected (ND) or not tested (NT) by RT-PCR are present below the lower horizontal black line. Circles represent samples that were SARS-CoV-2 tested by RT-PCR, and SARS-CoV-2 RNA was not detected by ISH in FFPE. Triangles represent samples that were SARS-CoV-2 tested by RT-PCR, and demonstrated positive detection of SARS-Co-2 RNA by ISH. Diamonds represent samples that were not tested (not available) by RT-PCR, and SARS-CoV-2 was positively detected by ISH on FFPE tissue. Squares represent samples in which SARS-CoV-2 tested by RT-PCR, however FFPE tissues was not tested (not available) for ISH. Stars represent cases in which tissue was unavailable for both SARS-CoV-2 RT-PCR and ISH.

The initial detection of SARS-CoV-2 infection was from deep nasopharyngeal swabs and fresh lung tissue by RT-PCR from five necropsied mink from two farms. After the initial SARS-CoV-2 diagnosis, multiple additional tissues from four necropsied animals (from the 20 described above) were collected in TRIzol for further investigation of viral tissue distribution by RT-PCR (designated mink 1–4). These four mink were randomly chosen for tissue collection based on the presence of grossly evident pulmonary edema and good post-mortem tissue quality (minimal autolysis). Viral RNA was detected in many tissues from multiple mink (Fig 3). Tissues where SARS-CoV-2 viral RNA was consistently detected between animals included nasal turbinates and lung, where nasal turbinates had a lower cycle threshold detectability than lung. Other tissues where viral RNA was detected included the retropharyngeal lymph node (3/4 mink), tracheobronchial lymph node (3/4 mink), squamous tissue from the distal nose (3/4 mink), paw pads (3/4 mink), and brain (3/4 mink). Detectible viral RNA was observed in other tissues with less frequency between animals. Once it was identified that nasal turbinates from two of the initially sampled mink (mink 3 and 4) had very low Ct detectability by RT-PCR (interpreted as a high viral load), RT-PCR was performed on formalin-fixed paraffin embedded (FFPE) sections of nasal turbinates from two additional randomly selected mink from the initial 20 that were necropsied (mink 5 and 6), where SARS-CoV-2 RNA was also detected. Associations between the additional microscopic lesions listed in the pathology results sections (such as enterocolitis or meningitis) and detectable SARS-CoV-2 were not investigated by PCR or *in situ* hybridization.

### Virus sequence analysis

Following initial viral detection, two mink viruses from farm A, four from farm B, and two from Farm C were submitted to the National Veterinary Services Laboratories (NVSL) in Ames Iowa for whole genome sequencing (WGS). Sequences were identical between animals from the same farm, with the exception of some ambiguous base calls and one sequence from farm A had an A38S-M mutation. Mutational analysis was performed using the GISAID EpiFlu Database CoVsurver, and analysis of hCoV-19 at https://www.gisaid.org/epiflu-applications/covsurver-mutations-app and CoV-GLUE application at http://cov-glue.cvr.gla.ac.uk/#/home. The SARS-CoV-2 viral sequences from all mink were in GISAID clade GH. One of the two mink from farm A had mutations at T85I-NSP2, S1205L-NSP3, G37E-NSP9, P323L-NSP12, T91M-NSP15, D614G-spike, N501T-spike, Q57H-NS3, H182Y-NS3, and T205I-N as compared to hCoV-19/Wuhan/WIV04/2019. These mutations were identical to those found in the closest GenBank match MW474212:SARS-CoV-2/human/USA/WA-S3043/2020. Synonymous SNPs from this Farm A isolate included C1059T, C3037T, C6336T, G12795A, C14408T, C20930T, A23064C, A23403G, G25563T, C25936T, C28887T, were also identical to those of MW474212 from a human in Washington State. The second farm A virus and all four farm B viruses had the same mutations as farm A, with additional mutation A38S-M, and additional mutation Q289H-N in Farm B viruses only. The two farm C viruses had the same mutations as the initial farm A, with additional mutations K113T-spike and V187I-NSP13. A query of all GISAID sequences from *Neovison vison* viruses revealed that 95.4% had mutations D614G and N501T. 69.1% had mutation H182Y. Seven non-synonymous mutations found in mink from this study were not found in any of the 955 mink sequences deposited in GISAID. These unique mutations included S1206L-NSP3, V1871I-NSP13, T91M-NSP16, K113T-spike, A38S-M, T2051-N, and Q289H-N. included S1206L-NSP3, V1871I-NSP13, T91M-NSP16, K113T-spike, A38S-M, T2051-N, and Q289H-N. The 8 whole viral genome sequences from this study were deposited into the GISAID database by NVSL as EPI_ISL_2896203, EPI_ISL_2896204, EPI_ISL_2896205, EPI_ISL_2896206, EPI_ISL_2896207, EPI_ISL_2896208, EPI_ISL_2896209, and EPI_ISL_2896210. See Table 1 for all SNPs and aa mutations.

**Table 1:** Mutations identified in the SARS-CoV-2 genome of Utah mink isolates. SNPs and nonsynonymous mutations identified. Amino acid (AA) and codon numbering is relative to Wuhan-Hu-1. Percentages of non-synonomous mutations found in *Neovison vision* data from 955 GISAID whole genome sequences is indicated. * = mutations unique to this study.

Gene	Mutation Farm A	Mutation Farm B	Mutation Farm C	Mutation type	% found in mink
NSP2	T85I	T85I	T85I	AA substitution	12.1%
NSP3	S1206L	S1206L	S1206L	AA substitution	0.8%*
NSP9	G37E	G37E	G37E	AA substitution	19.5%
NSP12	P323L	P323L	P323L	AA substitution	95.4%
NSP13			V187I	AA substitution	0.2%*
NSP16	T91M	T91M	T91M	AA substitution	0.8%*
Spike	N501T	N501T	N501T	AA substitution	12.3%
Spike	D614G	D614G	D614G	AA substitution	95.4%
Spike			K113T	AA substitution	0.2%*
NS3	Q57H	Q57H	Q57H	AA substitution	18.7%
NS3	H182Y	H182Y	H182Y	AA substitution	69.1%
M		A38S		AA substitution	0.4%*
N	T205I		T205I	AA substitution	0.8%*
N	Q289H (1 of 2)	Q289H		AA substitution	0.5%*

### Distribution of viral RNA in tissues by ISH

**Fig 4:**
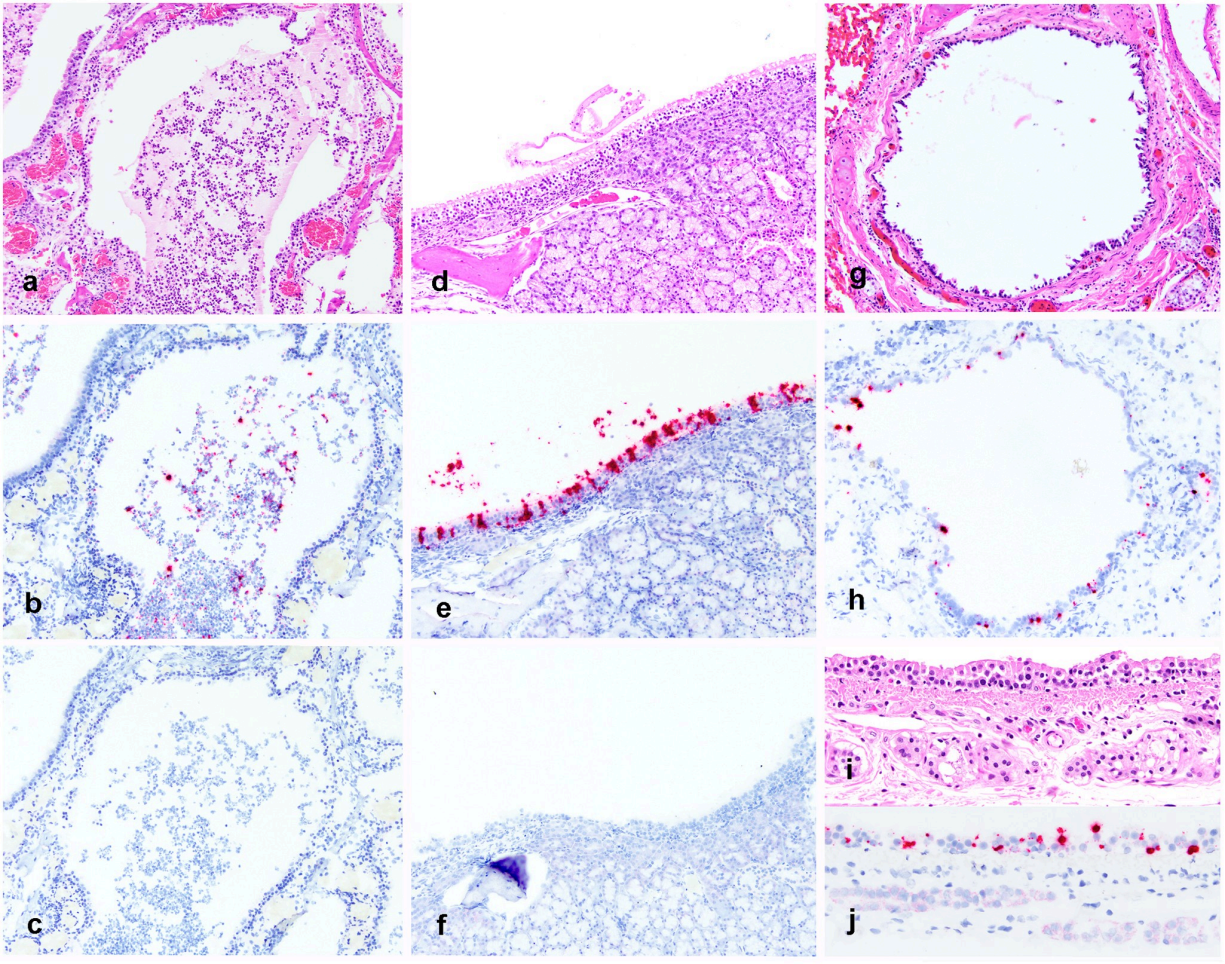
Detection of SARS-CoV-2 RNA in tissues by ISH. Fig A-C. Nasal turbinate samples from SARS-CoV-2 infected mink 5. A. H&E image of suppurative and histiocytic rhinitis filling the nasal passage. B. Detection of SARS-CoV-2 RNA in the nasal exudate. C. No detection of FIPV RNA in nasal turbinate of SARS-CoV-2 infected mink (negative control). D. Nasal turbinate samples from SARS-CoV-2 infected mink 5 demonstrating mild rhinitis in the caudal nasal passage and mild disorganization of respiratory epithelial cells E. Detection of SARS-CoV-2 viral RNA within respiratory epithelial cells of mink 5. F. No detection of FIPV RNA in nasal turbinate epithelial cells of SARS-CoV-2 infected mink (negative control). Fig G-H Lung samples from SARS-CoV-2 infected mink 1. G. H&E image of bronchus with attenuation and multifocal loss of respiratory epithelial cells. H. Detection of SARS-CoV-2 viral RNA within respiratory epithelial cells of the bronchus. Fig I-J Trachea samples from SARS-CoV-2 infected mink 1. I. H&E image of trachea with mild attenuation and multifocal disorganization of respiratory epithelial cells. J. Detection of SARS-CoV-2 viral RNA within respiratory epithelial cells of the trachea.

SARS-CoV-2 RNA was detected by chromogenic *in situ* hybridization in multiple FFPE tissues (Fig 3). The nasal turbinates and nasal passages of the two mink in which tissues were available for evaluation had abundant positive staining for viral RNA in the suppurative and catarrhal exudate within the nasal passages (Fig 4A–4C), as well as in the respiratory epithelial cells of the most caudal nasal passage overlying nasal mucous glands (Fig 4D–4F). In 4/4 mink there was positive detection of viral RNA in pulmonary bronchial epithelial cells or multifocally within the interstitium (Fig 4G and 4H). Distinct SARS-CoV-2 staining was not observed in the inflammatory cells of the pulmonary vasculitis and perivasculitis. There was also positive detection of viral RNA in the tracheal epithelial cells of one mink 1 (Fig 4I and 4J). Other tissues where viral RNA was observed included the most superficial surface of the distal squamous nose (2/4 mink), the surface of the tongue (1/4 mink), and very little detection in the lumen of the colon (2/4 mink) and small intestine (1/4 mink). Thyroid gland, adrenal gland, eye, ovary, uterus and pancreas were examined from 1 mink by ISH in which viral RNA was not detected. Positive and negative tissue and reagent controls, as described in Materials and methods, performed as expected. In short, viral RNA was readily detected in FFPE tissues from an FIPV-infected domestic cat with identical tissue pretreatments to the mink tissues.

## Discussion

In this report we show that mink are highly susceptible to SARS-CoV-2 infection, and that infection can be associated with high mortality in a natural farm production setting. Furthermore, we describe the pathology, tissue and cellular distribution of virus, and viral genetics in infected animals. Since the emergence of SARS-CoV-2, mink have been the only animal species identified to develop significant disease associated with infection in the United States. Infection in this case was suspected to be the result of reverse zoonotic transmission (from humans to mink), similar to other SARS-CoV-2 infections reported in animals, however this was not definitively determined in this investigation. The occurrence of disease in many animals on multiple farms is highly suggestive of significant mink-to-mink transmission, which raises concern regarding propagation of viral mutants that could have greater fitness and virulence. In a recent Denmark investigation, SARS-CoV-2 infected mink, many that were asymptomatic, were suggested to serve as transmission vectors of a new mutated strain of virus to humans [33]. Our investigation of the viral molecular phylogeny of SARS-CoV-2 mutations described herein did not identify the mutations associated with viral transmission in the aforementioned Danish study. We did not investigate the development of mutations to the SARS-CoV-2 genome over the duration of this outbreak, however such an epidemiological study would be interesting.

The outbreaks of SARS-CoV-2 in farmed mink in April 2020 in the Netherlands and Denmark showed robust viral transmission, similar to what we have described here, however mortality rates in our Utah outbreak were much higher (up to 55%), compared to the Netherlands outbreak, which reported 2.4% mortality at greatest, and the Danish outbreak, which showed minimal clinical disease and mortality [32,33,39]. Such a substantial difference in mortality may be due to the population of mink considered in the mortality rate (only adults were considered in this case, while young animals may have been included in the Netherland report), or other reasons such as differences in housing and management, comorbidities (such as infection with Aleutian Disease virus), or viral virulence.

Our pathologic investigation demonstrated that the most consistent and significant changes were observed in the respiratory tract, and death was attributed to pulmonary failure and edema. Histologically, the respiratory changes were typical of viral interstitial pneumonia with alveolar damage, consistent with the pulmonary histopathology described in the Netherlands mink outbreak [39]. The Netherland’s mink outbreak investigation revealed viral RNA and protein present in multiple organ systems, most consistently detectable in respiratory system [32,39]. One interesting histopathologic finding of note in our case not described in the Netherland outbreaks was the presence of perivascular mononuclear inflammatory cells, edema and rare vascular wall fibrinoid necrosis (vasculitis), which has been described in humans and experimentally infected ferrets [15,40–42]. In a recent report of describing the pulmonary pathology from human Covid-19 deaths, a key histologic feature of was the presence of increased numbers of perivascular T-lymphocytes (termed pulmonary vascular endothelialitis), though this feature did not definitively distinguish it from influenza pneumonia [42]. Given some of the striking similarities between the pulmonary histopathology of SARS-CoV-2 infected mink and humans, and abundance of virus in the upper respiratory tract between species, mink should be considered as a possible natural disease model of human Covid-19 disease. However, the vascular changes observed in the Utah outbreak reported herein could also be due to co-infection, particularly by Aleutian Disease virus, which was known to cause disease within the affected farms in the past.

The finding of severe suppurative and catarrhal rhinitis observed in the infected Utah mink was also an interesting finding. Rhinitis has been described in association with SARS-CoV-2 infection in experimentally infected cats, but the nature of the inflammation was described as mononuclear rather than suppurative [15]. Examination of the nasal conchae in the Netherlands mink report revealed swelling and degeneration of epithelial cells with diffuse loss of cilia and mild inflammation, which wasn’t further characterized [39]. In any case, significant differences were observed in the nasal inflammation between these two outbreaks, which should be addressed in future investigations.

The tissue distribution of virus investigated by RT-PCR reported herein revealed consistent detection of viral RNA in upper and lower respiratory tissues. Interestingly, RT-PCR also detected viral RNA in the brain, spleen, and various lymph nodes of multiple mink. By ISH, viral RNA was localized to respiratory epithelial cells of nasal turbinates, trachea and bronchi with multifocal detection in the pulmonary interstitium of some mink. These cellular localization findings are similar to the Netherlands investigation, which demonstrated viral antigen in epithelial cells in the same locations [39]. In experimentally infected cats, ferrets and Syrian hamsters the distribution of viral antigen localization was similar [15,21]. In this case we also identified viral RNA present on superficial epithelial surfaces of the distal nose, tongue and rarely within the lumen of the intestines by ISH, which we interpret as likely shedding from the infected nasal passage and passive surface accumulation or ingestion. This finding is interesting and may suggest that infectious virions are present on superficial epithelial surfaces and are potential sources of viral transmission. Detection of intact infectious virions would be necessary to prove this hypothesis.

There were discrepancies in the tissue distribution of viral RNA as detected by RT-PCR and ISH in this report, which warrants further investigation. These differences could be due to a greater detection sensitivity by RT-PCR, contamination of samples during collection at necropsy and detection by RT-PCR, viremia with rare or inconsistent detection in various tissue systems or circulating RNA. In a recent study investigating the utility of RNA-ISH, immunohistochemistry and RT-PCR in humans infected with SARS-CoV-2, ISH had a sensitivity and specificity of 86.7% and 100% respectively compared to RT-PCR [43]. Additionally, they report that RT-PCR and ISH consistently demonstrated the presence of viral RNA within pulmonary tissues, where viral RNA was not detected in any extrapulmonary tissues by either method. Another likely contributor to the differences we report here may be due to the small sample size of mink investigated, which is considered a limitation of this report.

Omitting 42 ambiguous bp reads in the stable NSP-9 region, the initial whole viral genome sequences from the farm A mink isolates examined as part of our Utah mink farm outbreak were 100% identical with three human SARS-CoV-2 GenBank accessions from Washington State, MW474211, MW474212, and MW474111, and mutations discovered via GISAID analysis are identical between the mink isolates and these three human isolates. GISAID differentiates COVID-19 into three major clades: Clade S, Clade V and Clade G (originally prevalent in North America, Asia/Europe, and Europe, respectively), based on NS mutations at NS8_L84S, NS3_G251V and S_D614G, respectively [44]. The G clade was subsequently divided into GR clade containing N_203–204: RG>KR and GH clade with NS3_Q57H aa substitutions [45]. The Utah mink isolates fall into clade GH. Analysis via the CoV-GLUE website at http://cov-glue.cvr.gla.ac.uk/#/home classifies these viruses in the B.1 lineage of the Rambaut et al. lineage system [46,47]. This lineage originally comprised the Italian outbreak before spreading to Europe and other parts of the world.

Fourteen nonsynonymous sequence mutations were identified in the SARS-CoV-2 genome from the Utah mink isolates we report herein. The polyprotein ORF1ab T85I-NPS2 mutation is most common in the USA (56% of phase 2 viruses) and has spread to at least 37 countries during phase 2 of the pandemic [48]. The P323L-NSP12 mutation in the viral polymerase gene coevolved with the D614G-spike mutation also present in this mink strain to become the most prevalent variant in the world, and this combination is present in 95.4% of whole virus genomes from mink to date. The G614 variant of the spike is more infectious than the original Wuhan D614 variant. Success of the P323L/ G614 variant suggests that the P323L mutation adds to the virulence of the G614 spike variant, although without increasing patient mortality. [49]. In addition to the highly prevalent D614G mutation, the mink isolate had a N501T spike mutation, which is uncommon in humans. This unncommon spike RBD N501T mutation from Utah mink has been found in four emergences within three lineages of mink samples [50]. GISAID reports that this mutation is related to host change and antigenic drift. N501T is located in the Receptor Binding Domain (RBD) of the spike glycoprotein, resulting in a moderate increase in ACE2 binding [51,52]. The NS3_Q57H mutation is common in the USA and is predicted to be deleterious [53]. S1205L-NSP3, T91M-NSP15, H182Y-NS3, Q289H-N, and A38S-M are rare mutations of unknown significance. The Utah mink did not share other spike RBD mutations Y453F and F486L, which are common in U.S. mink, nor did they have any of the common mutations reported from other mink throughout the world. These included five nsp2 aa substitutions (E352Q, A372V, R398C, A405T, and E743V), four in the nsp3 papain-like proteinase domain (P1096L, H1113Y, I1508V, and M1588K) one in the nsp5 3C-like proteinase domain (I3522V), one in the nsp9 RNA/ DNA binding domain (G4177E or R) one in the nsp15 poly(U) specific endoribonuclease domain (A6544T), two in the nsp12 RNA-dependent RNA polymerase domain (M4588I and T5195I), and two in the nsp13 helicase domain (I5582V and A5770D) [54]. With the exception of the common D614G mutation, the Utah mink have none of the multiple spike protein changes found in variants of concern, which the CDC currently lists as WHO label alpha, beta, delta, and gamma strains (Pango lineages B.1.1.7, B.1.351, B.1.617.2, and P.1).

In conclusion, our results indicate that mink are susceptible to SARS-CoV-2 infection. Infected animals suffer from severe respiratory disease, similar to that which has been described in humans, as well as other experimentally infected animals. Further investigations should focus on investigating the immunology and vascular pathology associated with the development of disease in mink to potentially extrapolate findings for human health and other animals. The Utah mink SARS-CoV-2 strain is unique among mink and other animal strains sequenced to date. Identical strains found in Washington state humans may reflect zooanthroponosis, and to date there is no evidence that viruses adapted to mink will impact human SARS-CoV-2 evolution. However, monitoring of mutations located within the RBD of the SARS-CoV-2 spike protein in mink is important for studying viral evolution and host-adaptation. Between August 2020 and the end of January 2021 the N501T mutation increased in frequency of sequenced isolates in the United States from.01% to.30%, similar to the increase in N501Y mutations. Lastly, strict biosafety measures are warranted on mink farms to decrease viral transmission between animals and risk of transmission to humans, as well as decreasing animal losses due to SARS-CoV-2 infection.

## Materials and methods

### Ethics statement

In most cases animals died acutely due to natural infection, and less commonly were euthanized by cervical dislocation when humane euthanasia was warranted according to Fur Commission USA standards. The mink were housed as distinct separate, private operations that fall outside of the IACUC approval required at universities. All farms were members of the Utah Fur Breeders Association, which is under the Fur Commission USA and all members follow standard guidelines for the operation of mink farms in the United States, which includes best practices for care, biosecurity and euthanasia. Investigation of any human impact or infections associated with this outbreak was out of the scope of this study.

### Pathology

Deceased mink were submitted to the Utah Veterinary Diagnostic Laboratory for investigation of the cause of death. At necropsy all body systems were examined by an ACVP-board certified anatomic pathologist (TB) and one anatomic pathology resident (MC) at the Utah Veterinary Diagnostic Laboratory. A full complement of tissues were collected from twenty mink and fixed in 10% neutral buffered formalin. Formalin fixed tissues were dehydrated in ethanol, embedded in paraffin wax, sectioned at 4 μm, and stained with hematoxylin and eosin using standard histochemical techniques. For SARS-CoV-2 RT-PCR, an extended list of tissues were collected and placed into TRIzol Reagent (ThermoFisher Scientific, Waltham, MA); Oronasal swabs were placed in viral transport medium (PrimeStore MTM; LongHorn Diagnostics).

### Swab sample extraction method

Total nucleic acid was extracted from samples in 1 mL of PrimeStore MTM [LongHorn Diagnostics] using MagMAX-96 Viral RNA Isolation Kit, per the manufacturer’s instructions.

### Tissue sample extraction method

RNA was extracted from fresh tissue samples in TRIzol Reagent and formalin fixed paraffin embedded tissues using TRIzol reagent [ThermoFisher, Waltham, MA 02451], per the manufacturer’s instructions. FFPE tissues were cut in 10um sections and heated at 65°C for 10 minutes in TRIzol prior to RNA extraction using MagMAX-96 Viral RNA Isolation Kit, per the manufacturer’s instructions.

### RT-PCR conditions

Reverse transcriptase (RT) real-time PCR to the SARS-CoV-2 RNA-dependent RNA polymerase gene (RDRp) was performed as previously described using primers SARS-CoV-2 primers RdRp_SARSr-F2 5’-GTGARATGGTCATGTGTGGCGG-3’ and COVID-410R 5’-CCAACATTTTGCTTCAGACATAAAAAC-3’ [55], using TaqMan Fast Virus 1-Step Master Mix Kit [Thermo Fisher]. RNA amplification was done using ABI 7500 Fast (ThermoFisher, Waltham, MA 02451). Controls included positive extraction control (RdRp_GATTAGCTAATGAGTGTGCTCAAGTATTGAGTGAAATGGTCATGTGTGGCGGTTCACTATATGTTAAACCAGGTGGAACCTCATCAGGAGATGCCACAACTGCTTATGCTAATAGTGTTTTTAACATTTGTCAAGCTGTCACGGCCAATGTTAATGCACTTTTATCTACTGATGGTAACAAAATTGCCGATAAGTATGTCCGCAATTTAC, negative extraction control (PCR water), positive amplification control (SARS-CoV-2 whole genome RNA), and negative amplification control (No template control). Graphs and tabular Ct results were reviewed on the ABI 7500 program. Unknown samples were considered positive if they rose above the threshold before cycle 45. All others were considered negative.

### Whole genome sequencing

Libraries for the whole genome sequencing were generated using the Ion AmpliSeq Kit for Chef DL8 and Ion AmpliSeq SARS-CoV-2 Research Panel (Thermo Scientific, Waltham, MA). Libraries were sequenced using an Ion 520 chip on the Ion S5 system using the Ion 510 & Ion 520 & Ion 530 Kit. Sequences were assembled using IRMA v. 0.6.7 and visually verified using DNAStar SeqMan NGen v. 14. Mutational analysis was performed using the GISAID EpiFlu Database CoVsurver: Mutation Analysis of hCoV-19 at https://www.gisaid.org/epiflu-applications/covsurver-mutations-app.

### Visualization of genomic material in tissues

In parallel with the collection of tissues collected in TRIzol for RT-PCR investigation, duplicate sections of those tissues were collected in formalin to be used for ISH from mink 1–4. *In situ* hybridization utilized RNAscope (Advanced Cell Diagnostics, Hayward, CA) technology to visualize the presence and location viral RNA in tissues harvested from infected mink. A set of anti-sense SARS-CoV-2 specific RNA probes comprised of 20 Z pairs targeting nucleotides 21,631–23,303 of the spike viral glycoprotein gene (Genbank accession number NC_045512.2) was developed by Advanced Cell Diagnostics (ACD) and performed as previously described [56]. This assay was performed according to manufacturer’s protocols for RNAscope 2.5 HD Red Detection Kit (ACD) with the following specific conditions. Fresh tissues from four SARS-CoV-2-positive and two SARS-CoV-2 negative mink were fixed in 10% buffered formalin for 48 hours, embedded in paraffin wax, and sectioned at 4um on positively charged glass slides. SARS-CoV-2 mink tissues were those that were present in the UVDL archive prior to the emergence of the SARS-CoV-2 pandemic. Samples were slowly submerged in lightly boiling Target Retrieval Solution (ACD) for 15 minutes, followed by application and incubation of Protease Plus (ACD) at 40°C for 20 minutes. A probe specific for a feline infectious peritonitis virus (FIPV) RNA also generated by ACD as positive and negative controls. FFPE tissues from a domestic cat with peritonitis due to FIPV-infection was used as positive assay control. Additionally, these FIPV-infected tissues, FFPE intestinal tissue from a bovine calf infected with bovine corona virus (confirmed by PCR), a coronavirus positive calf trachea and nasal turbinates from a domestic cat prior to the SARS-CoV-2 pandemic all were utilized as negative tissue controls and stained with the SARS-CoV-2 probe to investigate any cross-reactivity to these other coronaviruses and non-specific reactivity.

There was detection of viral RNA in inflamed splenic tissue from an FIPV infected cat, which served as a positive assay control for performance and presence of RNA in FFPE handled similar to the mink tissues in this study. No viral RNA was detected (no cross-reactivity) in the negative control slides handled similarly to the mink tissues. These negative controls included applying the SARS-CoV-2 probe to tissues (lung, lymph node, small intestine and colon) from one healthy adult mink that died of crush injuries prior to the emergence of SARS-CoV-2, spleen from an FIPV-infected cat, intestines and trachea from a bovine calf infected with bovine coronavirus, and nasal turbinates from a cat with suppurative rhinitis collect prior to the emergence of SARS-CoV-2. Additionally, there was no FIPV detection when this probe was applied to the SARS-CoV-2 positive mink nasal turbinates and lungs (Fig 4C and 4F).
